# Study of the Thyroid Profile of Patients with Alopecia

**DOI:** 10.3390/jcm12031115

**Published:** 2023-01-31

**Authors:** Adelina Popa, Mara Carsote, Dragos Cretoiu, Mihai Cristian Dumitrascu, Claudiu-Eduard Nistor, Florica Sandru

**Affiliations:** 1 Department of Dermatovenerology, “Carol Davila University” of Medicine and Pharmacy & “Elias” University Emergency Hospital, 011461 Bucharest, Romania; 2 Department of Endocrinology, “Carol Davila” University of Medicine and Pharmacy & “C.I. Parhon” National Institute of Endocrinology, 011461 Bucharest, Romania; 3 Department of Cellular and Molecular Biology, and Histology, Faculty of Medicine, “Carol Davila” University of Medicine and Pharmacy & National Institute for Mother and Child Health Alessandrescu-Rusescu, 011461 Bucharest, Romania; 4 Department of Obstetrics and Gynaecology, “Carol Davila” University of Medicine and Pharmacy & University Emergency Hospital, 011461 Bucharest, Romania; 5 Department 4–Cardio-Thoracic Pathology, Thoracic Surgery II Discipline, “Carol Davila” University of Medicine and Pharmacy & Thoracic Surgery Department, “Carol Davila” Central Emergency University Military Hospital, 011461 Bucharest, Romania

**Keywords:** thyroid, alopecia, thyroiditis, antibodies, endocrine, dermatology, hair, telogen effluvium

## Abstract

Thyroid hormones are required for the physiological growth and maintenance of hair follicles. We aim to study the thyroid profile of patients with alopecia. This is a narrative review. PubMed literature was searched from 2013 to 2022. We followed different types of alopecia: alopecia areata (AA), androgenic alopecia in males and females, telogen effluvium (TE), frontal fibrosing alopecia (FFA), lichen planopilaris, and alopecia neoplastica (AN). AA shares a common autoimmune background with autoimmune thyroid diseases, either sporadic or belonging to autoimmune polyglandular syndromes. Some data suggested that AA is more severe if thyroid anomalies are confirmed, including subclinical dysfunction or positive antithyroid antibodies with normal hormone values. However, routine thyroid screening for patients with AA, if the patients are asymptomatic from a thyroid point of view and they have negative personal and family history of autoimmunity, remains controversial. TE, apart from the autoimmune type, associates thyroid anomalies of a hormonal assay (between 5.7% and 17%). FFA, mostly a postmenopausal entity (however, not exclusive), associates a higher prevalence of thyroid conditions (up to 50%) than the general population. However, these might have an age-dependent pattern, thus the association may be incidental since there are a limited number of studies. Overall, alopecia remains a very challenging condition for patients and physicians; a multidisciplinary team is required to improve the outcome and quality of life. The common autoimmune background is suggestive of some types of alopecia and thyroid disorders, yet, the underlying mechanisms are still a matter of debate. AA, TE, FFA, LPP, and, potentially, female pattern hair loss have been found to be connected with thyroid entities, thus a state of awareness from a dual perspective, of trichology and endocrinology, is helpful.

## 1. Introduction

Human hair plays a major role in social communication and different behaviours, and its loss (alopecia) is typically associated with a decreased sense of personal wellbeing, low self-esteem, and social disengagement. Despite recent progress, alopecia remains a challenging condition due to its complexity, multidisciplinary implications, and difficulties in management in many situations. It is characterized by diffuse or patches of hair loss, total hair loss of the scalp (alopecia totalis), and/or of the entire body (alopecia universalis). The pathogenesis is incompletely described, however, a connection to immune and cytokines status, as well as to the endocrine field, including the thyroid domain, should be taken into consideration [1,2,3,4,5].

### 1.1. Thyroid Hormones (THs) and Hair Follicles

THs are required for the physiological growth and maintenance of hair follicles, suggesting that hair loss could be a sign of a thyroid disorder [1]. When it comes to the thyroid panel in relationship to alopecia, there are several levels of approach. On one hand, the role of THs at the level of the skin may be stratified in terms of endocrine, paracrine, and autocrine roles. On the other hand, associated pathologies to the thyroid diseases themselves might play a role in the development of alopecia as does gonadal status, including androgens levels, insulin resistance, metabolic complications, vitamin D deficiency, etc., as well as the connection with thyroid autoimmunity [1,2,3,4,5,6,7,8,9,10]. TH actions at a peripheral level in terms of receptors (TR) are related to skin and hair follicles, acting as endogenous modulators of dermal proliferation and local inflammation. KO mice for TRα1 and TRβ present a decreased level of keratinocyte proliferation (at the interfollicular epidermis) by involving a down-regulation of cyclin D1 and an upregulation of cyclin-dependent kinase inhibitors p19 and p27, respectively [3]. Lack of TRs might influence the bulge stem cells responsible for hair cycling (at the level of hair follicles). Further on, the dysfunction of these mentioned cells underlines abnormal Smad signaling and beta-catenin pathways [4]. These findings suggest that the dermal actions of THs are part of the local environment concerning hair follicles [3,4].

### 1.2. Alopecia: From General Considerations to Thyroid Connections

In terms of epidemiological impact, androgenic alopecia, the most prevalent cause of hair loss in the general population, is followed by alopecia areata (AA) and telogen effluvium (TE), affecting approximately half of all men and women. It is typically described as a most probable genetically determined condition that results in permanent hair loss in both men and women. The SOX gene has been proposed as a master regulator of hair shaft cuticle development [4]. However, alopecia may be incidental to a certain thyroid entity, considering its high prevalence in some populations.

With respect to the autoimmunity background, the prevalence of thyroid illness in alopecia patients ranges from 8% to 28% depending on the studied population, the types of alopecia, the types of thyroid diseases, etc. [1]. Kasumagić-Halilović described a significant link between alopecia and thyroid autoimmunity, with a higher prevalence of antithyroid antiantibodies (25.7%) in alopecia patients when compared to healthy people (3.3%) [2]. AA, a particular immune-mediated chronic disorder with periodic relapses, causes nonscarring hair loss; about 2% of the general population develops the disease with cosmetic disadvantages, impairing quality of life. Hair follicles are retained in AA, with the possibility of regeneration. Although scalp hair follicles are the primary targets, extra-scalp involvement, which manifests as facial hair loss (eyebrows, eyelashes, beard) or body hair loss, is found in AA, being linked to other systemic disorders such as atopic, autoimmune, and rheumatoid diseases [6].

Alopecia in subjects with chronic autoimmune Hashimoto’s thyroiditis is either first referred to a dermatologist or the patient is already under endocrine surveillance. CD8+ positive lymphocyte infiltration, as found in chronic thyroiditis, is dependent on an interferon-mediated response, and this aspect is identified in other types of alopecia like frontal fibrosing alopecia (FFA). This represents a scarring lymphocytic alopecia which is accompanied by the absence of eyebrows, being reported in some cases of severe hypothyroidism, and generally, it remains of a less clearly understood cause [7,8,9,10,11]. Early-onset type I interferonopathy is caused by monogenic STING gain-of-function mutations, with symptoms ranging from catastrophic vasculopathy to moderate chilblain lupus. The underlying molecular processes of the varied phenotype-genotype connections are still unknown. Alopecia, photosensitivity, and thyroid dysfunctions are among the characteristics of STING-associated vasculopathy with onset in infancy (SAVI), also involving livedo reticularis, skin vasculitis, nasal septum perforation, face erythema, and bacterial infections [12]. An extremely rare type is alopecia neoplastica (AN), an uncommon form of cutaneous metastasis originating from visceral tumours, including thyroid cancer [13].

### 1.3. Aim

We aim to analyse thyroid profiles in individuals with different types of alopecia.

## 2. Methods

This is a narrative review. A PubMed literature search was conducted using the terms: “alopecia” and “thyroid” (or “thyroiditis”), from 2013 to 2022. We followed different types of alopecia: alopecia areata (AA), androgenic alopecia in males and females, telogen effluvium (TE), alopecia neoplastica (AN), frontal fibrosing alopecia (FFA), and lichen planopilaris (LPP), and included only clinically relevant studies (full-length papers and excluded case reports) that provided enough information on the dermatological issues in relation to thyroid diseases.

## 3. Different Types of Alopecia: The Link to the Thyroid Profile

### 3.1. Alopecia Areata (AA)

The cause of AA is unknown; however, genetic susceptibility, different types of autoimmunity, and, probably, stress are regarded as contributors [6,14]. AA is thought to be caused by a loss of immunological privilege and a subsequent attack on the follicle by CD8 + ve T cells [5]. It occurs in episodic (recurrent) patterns, with an incidence rate of 0.1–0.2%, respective of 7–30 cases per 1000 dermatological patients, and a lifetime risk of 1.7% [6,14]. The lesions are self-limiting, small sized, or, rarely, large with extensive spread out. The random clinical course is not correlated with age, gender, or race [6,14]. Patchy alopecia, alopecia reticularis, alopecia totalis, and alopecia universalis are regarded as clinical variants of AA [15].

In AA patients, autoimmune conditions, including Hashimoto’s thyroiditis, were found more frequent than in the general population [6,15]. A systematic meta-analysis performed by Lee et al. showed that autoimmune thyroid disease (ATD), including Graves’s disease and Hashimoto chronic thyroiditis, were more frequent in individuals with AA than in the controls. Patients with AA were more likely to have a thyroid dysfunction (OR = 4.36; 95% CI 1.19–15.99; prevalence of 12.5%), particularly subclinical hyperthyroidism (OR = 5.55; 95% CI 1.73–17.85; prevalence of 5.7%), and subclinical hypothyroidism (OR = 19.61; 95% CI 4.07–94.41; prevalence of 10.4%) [16].

Another meta-analysis by Kinoshita-Ise et al. in 2019 showed that anti-thyroid peroxidase antibodies (TPO-Ab) are more common in AA patients than in controls (OR = 3.58; 95% CI 1.96–6.53), as well as antithyroglobulin antibodies (TG-Ab) levels (OR = 4.44; 95% CI 1.54–12.75). The risk of having both positive TPO-Ab and TG-Ab in the same subject with AA is OR = 2.32 (95% CI 1.08–4.98), or having either one positive is OR = 6.34 (95% CI 2.24–17.93) compared to controls (AA free). TSH (Thyroid Stimulating Hormone)-receptor antibody (TR-Ab) prevalence is higher in AA patients (OR = 60.90; 95% CI 34.61–107.18) [17]. This association between AA and thyroid autoimmunity is confirmed, however, not with thyroid dysfunctions (subclinical or clinically manifested), as previously mentioned [16,17]. Despite including 17 studies (a total of 262,581 subjects and more than one million persons as the control group), longitudinal data were not consistent for clear conclusions. Screening for thyroid conditions in subjects with AA is particularly encouraged in those cases with severe phenotypes [17].

T lymphocytes cause hair destruction in AA, hence T lymphocytes and associated cytokines that infiltrate the areas around the hair follicles play a major part in the disease’s aetiology. Regulatory T cells are involved in the progression of AA and also of thyroid illness. HLA (Human Leucocytes Antigens) represents another link between AA and autoimmune thyroid disease. Some data showed that HLA-DQB1*03 is connected to both AA and antibodies-induced hypothyroidism. A genetic association study of HLA genes also found that the DRB1*15: 01 DQB1*06: 02 haplotype frequency was considerably greater in TR-Ab-positive individuals diagnosed with AA versus controls. The likelihood of AA patients associating hyperthyroidism or hypothyroidism is increased in comparison to controls (hyperthyroidism: OR = 1.43, 95% CI 0.36–5.57; hypothyroidism: OR = 4.07, 95% CI 1.95–8.49). Overall, among the 17 articles (N = 2850 patients with AA) the prevalence of any type of thyroid entity in terms of hormonal dysfunction and/or autoimmunity is higher than in the control (OR = 3.66, 95% CI 2.9–4.6) [18].

Dai et al. identified a bidirectional link between AA and thyroid illness in a nationwide population-based cohort study. The presence of thyroid conditions increases the risk of AA, while AA increased the risk of developing/presenting with thyroid disease. Interestingly, AA patients had a higher risk of acquiring nonautoimmune diseases, like toxic nodular goitre (aHR = 10.17; 95% CI 5.32–19.44), nontoxic nodular goitre (aHR = 5.23; 95% CI 3.76–7.28), and hormonal anomalies like thyrotoxicosis (aHR = 7.96; 95% CI 6.01–10.54), particularly Graves’ disease (aHR = 8.36; 95% CI 5.66–12.35), while Hashimoto’s thyroiditis was also statistically significantly more frequent (aHR = 4.35; 95% CI 1.88–10.04). Thyroid diseases seem prone to developing AA. The authors included 35,071 patients with thyrotoxicosis, 19,227 individuals with Graves’ disease, 5460 subjects with thyroiditis, and 3352 persons with Hashimoto’s thyroiditis (respectively, 1:10 matched controls). Patients with thyrotoxicosis had a noticeably higher risk of acquiring AA than healthy individuals (aHR = 9.29; 95% CI 7.11–12.14), as seen in Graves’ disease (aHR = 8.66; 95% CI 6.03–12.42), and thyroiditis (aHR = 6.42; 95% CI 3.15–13.11). However, there was no statistical significance for subjects with Hashimoto’s thyroiditis, as opposed to other mentioned studies [17,18,19,20].

Overall, 5% of AA patients have Hashimoto’s thyroiditis-related subclinical hypothyroidism, 8.9% of them have any thyroid dysfunction (statistically, significantly higher than control), and 17.7% of AA patients have positive TPO-Ab levels (twice more frequent than the general population) with a female predominance (female-to-male ratio of 6.7:1) which is also registered in autoimmune thyroid conditions regardless the presence of AA [20]. AA might associate a complex spectrum of comorbidities, and thyroid involvement should be taken into consideration among them [20,21,22]. For instance, one study reported a rate of 18.8% (N = 540 patients with AA, 68.5% females; *p* = 0.0004, OR = 2.84; 95% CI 1.55–5.18) [21].

**Figure 1:**
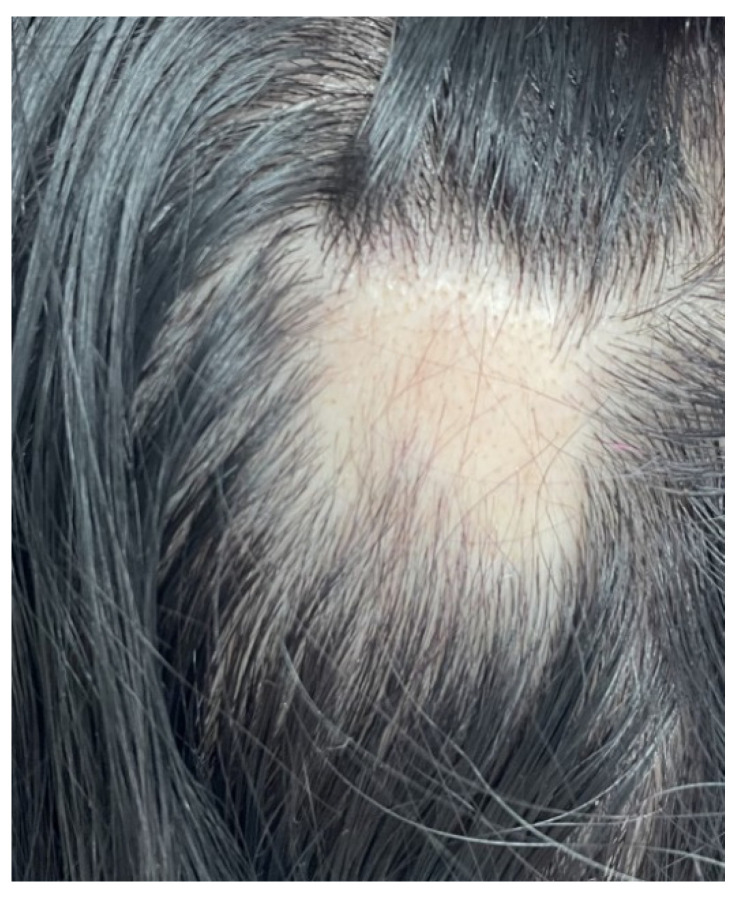
Patchy alopecia areata: a single, round-oval plaque with a diameter of approximately 4 cm, showing hair loss. This is a 54-year-old female patient diagnosed with Hashimoto’s thyroiditis (after alopecia onset) with normal thyroid function (TSH of 1 µUI/mL, normal between 0.5 and 4.5 µUI/mL, high TG-Ab of 129 UI/mL, normal < 4.11 UI/mL, and normal serum TPO-Ab of 3 UI/mL, normal < 5.6 UI/mL).

**Figure 2:**
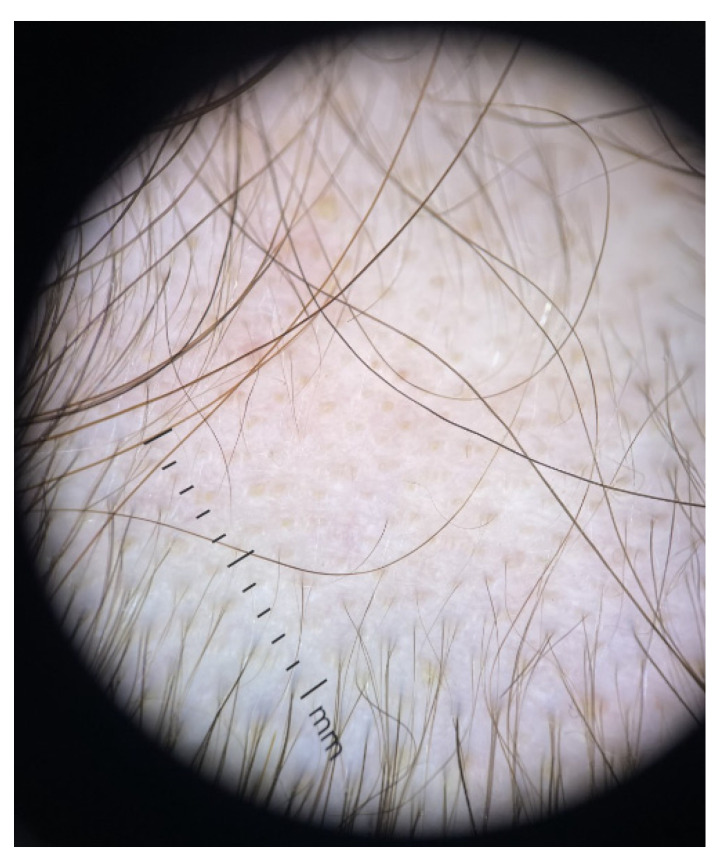
Trichoscopy aspect of alopecia areata; this is a 25-year-old female patient confirmed with Hashimoto’s thyroiditis and normal thyroid function (TSH of 2.27 µUI/mL, normal between 0.5 and 4.5 µUI/mL, high TPO-Ab of 109 UI/mL, normal < 35 UI/mL).

The association between AA and thyroid entities does not necessarily mean causality, and the topic is still open. If a selective subgroup of AA subjects is prone to develop endocrine anomalies at the thyroid level in terms of positive antibodies and/or abnormal hormonal panel and if these thyroid anomalies play an active role in developing a more severe form of AA is not unanimously confirmed (Figure 1 and Figure 2).

### 3.2. Androgenic Alopecia

Female androgenic alopecia, currently called female pattern hair loss (FPHL), is a nonscarring diffuse type caused by a progressive miniaturization of hair follicles and subsequent reduction of hair numbers, particularly in the central, frontal and parietal scalp regions, representing the most common cause of hair loss in adult females (general population) [23,24]. Some subgroups are at higher risk, especially seniors. One cross-sectional study from 2022 on menopausal women (N = 200, aged between 50 and 65 years) showed a 52.2% prevalence concerning FPHL which might be an additional contributor to impaired quality of life in menopause, thus the importance of awareness [25]. Whether thyroid disorders with an increasing prevalence in the aging female population are incidental or represent a pathogenic loop is not clear. We mention one prospective study on FPHL (aged between 20 and 88 years) that identified a hypothyroidism ratio of 31.25% in patients with FPHL without a correlation between FPHL severity and the presence of thyroid disease [26].

### 3.3. Male Pattern Alopecia

Male pattern alopecia (MPA) seems distinct from follicle shrinkage in FPHL. Some have suggested a connection with metabolic syndrome and insulin resistance and/or hypogonadism, which is not unanimously confirmed [27,28]. A descriptive study on 1232 men and women for 25 months identified 14.29% (N = 176) of them with MPA. The MPA subgroup showed a different prevalence of thyroid dysfunctions with respect to age; 6.3% in patients younger than 20 years, 17% in persons aged between 21 and 40 years, and 50% in individuals older than 40 years [29]. Early-onset androgenetic alopecia is related to low levels of testosterone and dehydroepiandrosterone, and testosterone replacement in hypogonadism might contribute to the reduction of antithyroid antibodies in these patients according to some authors [27,28,29,30]. One small study from 2021 in patients with autoimmune hypothyroidism requiring levothyroxine substitution included subjects with early onset MPA (N = 24) and without baldness (N = 24, two thyroid antibody-matched groups). Levothyroxine replacement had a more pronounced effect in correcting TH levels and antithyroid antibodies in males without alopecia, which suggests that MPA of this type might be a surrogate marker of response to TH replacement [30]. However, further evidence is necessary.

### 3.4. Telogen Effluvium (TE)

TE is a condition with a wide range of underlying elements. There are three main types of premature teloptosis, namely individual, collective, and premature entry into telogen. An additional fourth group includes different pathogenic entities, drug induced and dietary induced, and “autoimmune” TE. Despite this heterogenic panel, the majority of TE cases are actually of autoimmune causes [31,32,33,34].

Growing (anagen) human scalp hair follicles are particularly sensitive to mild fluctuations of serum THs as a result of the direct actions of T3 (triiodothyronine) and T4 (thyroxine) on this skin appendage. Thus, in patients with hypothyroidism, alopecia may occur, also embracing the aspect of prolonged TE, as well as dry, brittle, and dull hair shafts [32]. Despite the fact that THs stimulate hair matrix proliferation, hyperthyroidism can cause TE also [31,32,33,34]. The tensile strength of hair shafts from hyperthyroid subjects is also significantly diminished. In vivo effects of THs regulating hair development are proven in rats, lambs, mice, and humans. In human scalp hair follicles, TH-dependent signalling may prolong the anagen phase of hair growth and also modify the expression of certain keratins, increase hair matrix KC proliferation, and postpone the start of apoptosis-driven hair follicle involution (catagen). While THs have little effect on the creation of new hair shafts, T3 and T4 are responsible for increasing mitochondrial energy metabolism in human scalp hair follicles [32].

On a clinical level, we mention a retrospective study by Yorulmaz et al. that included 3028 patients with TE (92.3% females) with a median age of 26 years (IQR 20–37). The thyroid panel was the second most frequently performed lab test in patients initially admitted for TE to a dermatology unit (81.1%). However, THs were normal in 94.3% of the entire cohort, and only 3.9% experienced hypothyroidism, 1.8% had hyperthyroidism (a total ratio of 5.7% concerning the prevalence of any thyroid dysfunction). Of note, 74.4% of the patients with hyperthyroidism were older than 25 years [33].

Deo et al. conducted a study on 135 females (aged between 15 to 60 years) with different forms of alopecia and found that TE (62.2%) and FPHL (23.7%) were the most common. In the TE group, thyroid issues were detected in 17% (9.63% of all subjects had hypothyroidism, and 7.4% presented hyperthyroidism). Throughout the investigations starting from a dermatologic evaluation, 6.7% of patients were newly diagnosed with hypothyroidism and 3.7% with hyperthyroidism [34]. Whether the prevalence of thyroid function anomalies in TE are overlapping with the data regarding the general population is still a matter of debate. However, a certain selection of patients with TE to address endocrine concerns is necessary. Generally, the study we mentioned pointed out that TE is linked to the amount of TH in the bloodstream, and not to the autoimmune background with regard to the thyroid, as seen in other studies that predominantly included individuals with AA.

### 3.5. Alopecia Neoplastica (AN)

AN, an exceptional type of cutaneous metastasis at the scalp level, involves the scalp as the common site (that is explained by a higher blood supply in this area compared to other body regions). Also, the increased bloodstream explains the red/violaceous appearance [13,35]. Skin involvement might be a direct (local) extension of a tumour or a distant spreading (in advanced disease with a poor prognosis) [35]. Atrophy and/or loss of hair follicles are secondary to tumour invasion of the dermis and its desmoplastic reaction. However, the pathogenesis is less understood. The diagnosis might be difficult, especially in cases without evidence of an underlying malignancy or those mimicking AA of a small-size lesion [13,36,37]. Since the level of statistical evidence is low, we mention a systematic review of 123 individuals with histologically confirmed AN that identified 7.3% (9/123) of them with a thyroid malignancy; 66.7% (6/9) were females [13]. Of note, thyroid cancers (regardless of the type) and the associated scalp metastases are not common findings, thus the chapter on AN and thyroid neoplasia remains an open issue.

### 3.6. Frontal Fibrosing Alopecia (FFA)

FFA primarily affects menopausal women and causes progressive frontotemporal hairline regression, which may be accompanied by eyebrow hair loss. Recent data showed that FFA might be diagnosed in premenopausal women and even men as well [38,39,40,41,42,43,44]. The slow, gradual recession of the frontotemporal hairline with a full loss of hair and follicular apertures represents the hallmark of the condition. The presentation has been broadened to generalized body hair loss as well as particular skin findings, such as face papules and lichen planus pigmentosus [38,39,40]. Despite being asymptomatic, local accuses are sometimes described (as sensations of tightness, pain, or pruritus) [38,39,40,41]. The epidemiologic impact of the condition is less clear. However, the recent COVID-19 pandemic seemed to increase FFA prevalence due to stress-related effects and multiple social and environmental elements [38,42].

The connection with HLA class I allele-related susceptibility is one alternative theory. Others suggest the potential role of the Th1/JAK-STAT profile of inflammation [39,40,41,42]. One study (N = 932 patients with FFA, 96.9% females; disease duration of 3.7 years; mean age at onset of 55.6 years in women, respective of 60.6 years in males) showed that 31.4% had a thyroid dysfunction while nonthyroid comorbidities had a similar prevalence within the general population [39]. Another retrospective, a single centre study of 12 females with FFA (average age of 70.3 years; mean FFA duration of 5.67 years; all cases with post-menopausal self-reported onset) identified hypothyroidism as the most common comorbidity (58%) with a higher prevalence than the general population (6–10%), thus awareness is important [40]. A large observational, cross-sectional study included 490 patients with FFA (95% females; 84% of them were in menopause). The first elements of FFA were reported at various ages, between 15 and 89 years; however, FFA recognition might take up to 24 years. Thyroid dysfunction, the most prevalent comorbidity, was identified in 35% of women, and 13% of males [41]. A retro-prospective cohort study on 46 individuals with FFA and an additional 12 new cases showed a 15.7% rate of abnormal thyroid function [42]. The study of environmental factors as potential contributors in FFA revealed no significant association except for a history of thyroid disease (19% versus 7% in the control group, *p* < 0.05) [43]. A retrospective study from the Mayo Clinic (between 1992 and 2016) analysed seven males with FFA and none had any thyroid conditions [44]. Whether females with FFA are more likely to associate thyroid anomalies as opposed to males requires further research.

### 3.7. Lichen Planopilaris (LPP)

**Figure 3:**
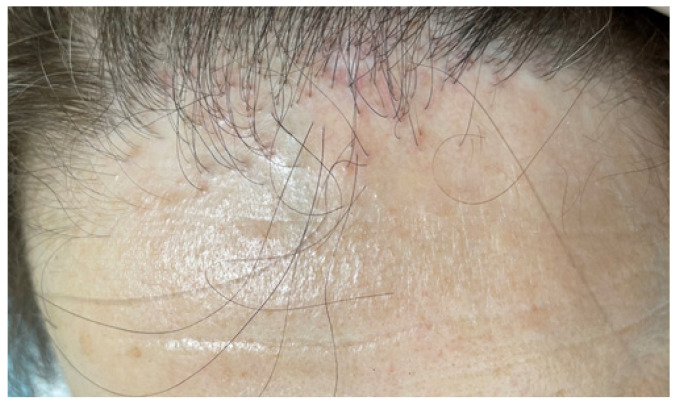
Lichen planopilaris clinical aspect: permanent hair loss located at the frontal level. No hair follicle openings can be seen in the area of hair loss. Scale and redness surround each hair follicle in the area of hair loss.

LPP, a rare lymphocytic-mediated illness that causes inflammation and scarring in the hair follicles, represents primary cicatricial alopecia of unknown cause, however, some suggested an autoimmune involvement, or an association with HLA, since the HLA DQB1 gene is linked to autoimmune noncicatricial alopecia susceptibility [45,46,47,48,49,50]. LPP affects the vertex of the scalp, accompanied by erythema, soreness, hyperkeratosis, and permanent scarring. Additionally, pruritus is reported [46,47,48,49,50] (Figure 3).

Although severe thyroid diseases might impact hair development, there is no clear pathogenic link between LPP and these diseases [47,48,49]. Despite thyroid entities not being a typical risk factors for LPP, some studies reported a higher prevalence in LPP subjects. For instance, one study (N = 166) identified a higher prevalence than controls (34% versus 11%). Hypothyroidism was the most frequent thyroid anomaly (29% of all). A history of hypothyroidism was found in 42% of the cohort, while 15% had the first identification of LPP [45]. Another study (N = 232 women with LPP) showed that 30.1% of them had any type of thyroid condition, however, again, hypothyroidism was the most important (23.3%) which was the single statistically significant thyroid pathology with regard to LPP [45,46]. Cantwell et al. reported in a series of 19 males that 15.8% had thyroid involvement [47]. The largest study we identified in LPP and thyroid data included 344 cases of LPP. The prevalence of thyroid conditions was statistically significantly higher than the control with respect to Hashimoto’s thyroiditis (6.3% *versus* 0%; *p* = 0.023), and hypothyroidism (24.3% *versus* 12.8%; *p* = 0.028) [48]. Other studies reported that 10.58% of females had hypothyroidism (N = 189 women with LPP) and 20.7% (N = 232), respectively [49,50].

## 4. Discussion

### 4.1. From Alopecia to Thyroid Anomalies

A large number of data connect alopecia with the field of thyroid conditions, in terms of abnormal function and/or positive antibodies. It is undeniable that severe changes in TH serum levels have an effect on human skin and appendages, both TRα and TRß being extensively expressed in the dermis, including in the hair follicles. While congenital hypothyroidism is associated with hair loss in infants, high telogen hair counts on the scalp might be a sign of hypothyroidism in adults [32,33,34]. Some data showed that AA seems more severe if thyroid anomalies are confirmed in one patient, including subclinical dysfunction or serologic positive profile for antithyroid antibodies with normal hormone values. However, routine thyroid screening in AA remains controversial if the patients are asymptomatic from an endocrine point of view (for instance, concerning the patients with alopecia who do not actually present typical signs, symptoms, and even complications of untreated hypothyroidism as somnolence, bradycardia, cardiac insufficiency, dyspnea, even coma of unknown cause, persistently dry skin, and slow intestinal transit) and they have a negative personal and family autoimmunity [51,52,53].

**Figure 4:**
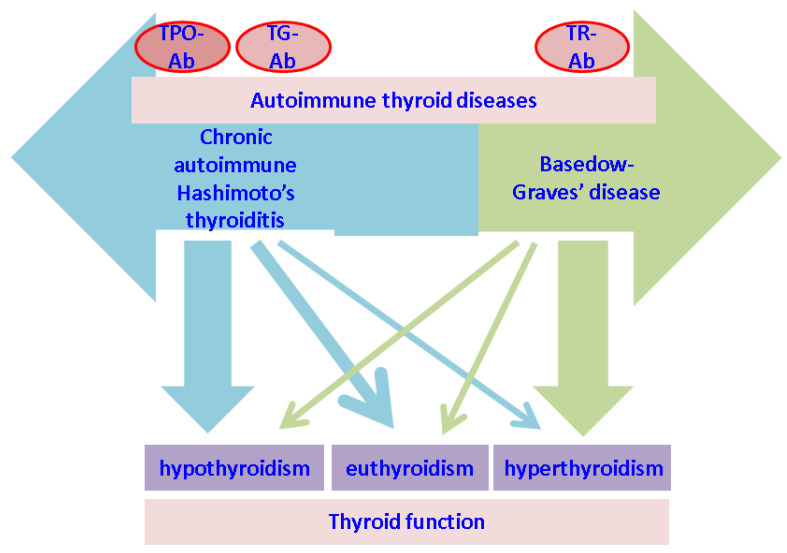
The relationship between thyroid hormonal profile and thyroid autoimmunity: Hashimoto’s thyroiditis and Graves’s disease belong to the spectrum of autoimmune thyroid diseases; most patients with Hashimoto’s thyroiditis have hypo or euthyroidism, but flare-up thyrotoxicosis might be present; Graves’s disease associates hyperthyroidism, however, normal thyroid function may be spontaneously or iatrogenic achieved while iatrogenic intervention (thyroidectomy, radioiodine therapy and anti-thyroid drugs) or copresence of blocking antithyroid antibodies causes hypothyroidism. Abbreviations: TPO-Ab = anti-thyroperoxidase antibodies; TG-Ab = antithyroglobulin antibodies; TR-Ab = TSH-receptor antibody.

Of note, the majority of thyroid involvement that has been studied in relation to alopecia involves anomalies of function (hypothyroidism or hyperthyroidism) and/or positive antithyroid antibodies, mainly TPO-Ab and Tg-Ab, which are consistent for Hashimoto’s thyroiditis and TR-Ab that offers the confirmation of Basedow-Graves’s disease. However, both autoimmune conditions are included in the spectrum of thyroiditis. A patient in either category of autoimmune thyroid condition might suffer from hyperfunction or hypofunction at a certain point in time, and also might present euthyroidism, either spontaneous or medically induced (for instance, by being treated with levothyroxine replacement in cases of hypothyroidism or with antithyroid drugs for Graves’ disease). Thus the clinical relevance of many studies (especially those cross-sectional) is limited concerning the association between the thyroid profile and alopecia [2,6] (Figure 4).

Deregulation of the immune system results in an immunological attack on the thyroid gland, which causes ATD [54]. Underlying the immune process in AA, peribulbar lymphocytes around the bulb region of anagen hair follicles are a tell-tale sign of active AA [55,56]. Natural killer group 2D (NKG2D) ligand in this area draws killer CD8 T cells to this infiltration [57]. Innate and acquired immunity are involved in AA [58,59]. The undulant pattern of autoimmunity is also reflected in the clinical pattern of AA. Multiple episodes of spontaneous regrowth are reported in a subject with AA. Within one year, the hair may return in 50–80% of patchy AA. Remission may be sustained, or the lesions are irreversible [60], which is why the AA impact of correcting thyroid dysfunction is still an open issue, and further interventional trials are needed. Autoimmunity is also involved in autoimmune TE, but generally, TE (which sometimes is clinically mistaken as AA) may associate with an abnormal hormonal profile regardless of the subtype [34,61,62,63,64,65,66].

### 4.2. Cicatricial Alopecia and Thyroid Status

Another chapter includes the thyroid profile in individuals with cicatriceal alopecias (FFA and LPP) [67,68,69,70]. FFA brings the menopausal panel as the main endocrine aspect, despite not being exclusively after menopause [71,72,73,74]. We still need evidence with regard to thyroid disease, including the mutual influence of thyroid anomalies and FFA [75,76,77,78,79,80]. The loss of the hair follicle’s immunological privilege would be the first step in the emergence of scarring alopecias [81,82,83,84]. Interferon may be the cause of this bulge immunological privilege collapse [85,86,87]. An important role in FFA is played by a Th1-biased cytotoxic T cell autoimmune response directed towards the hair follicles [88]. In the bulge region of LPP, downregulation of the hair follicle epithelial progenitor cell marker keratin 15 is found [89,90]. In FFA, and LPP, the lower melanocyte count found in the upper follicle indicates that the melanocytes of the hair follicle may be an antigenic target in FFA [91,92,93]. It has been suggested that the early causes of inflammation in LPP are the downregulation and abnormal action of the peroxisome proliferator-activated receptor (PPAR); PPAR being essential for sebocyte differentiation and maturation as well as lipid homeostasis [90,94,95]. Targeted ablation of PPAR in mice from follicular stem cells causes a scarring alopecia-like appearance [94,95,96,97,98].

Additionally, since the menopausal population is mainly affected by FFA, the role of androgen/oestrogens status has been incriminated [96,97]. The panel of postmenopausal dermatological and endocrine comorbidities might suggest a common pathogenic mechanism. For instance, subjects with FFA associate a higher risk of systemic lupus erythematosus, while FFA and vitiligo are reported to be associated with the same individuals with ATD [97,98,99].

Additionally, neutrophil-associated subtypes of primary cicatricial alopecia have been studied with regard to thyroid status. We identified one study from 2004 to 2013 that estimated an overall incidence of 6.1 (95% CI 5.62–6.6) per 100,000 person years and an overall prevalence of 20.93 (95% CI 17.97–23.86) per 100,000 person years, and logistic regression analysis confirmed a statistically significant association with thyroid diseases OR = 1.64 (*p* < 0.001) [100].

### 4.3. Polyglandular Autoimmune Syndrome

A very challenging condition from a multidisciplinary perspective, as it is not only dermatological or endocrine, is represented by autoimmune polyglandular syndrome type 1 (APS-1), a rare autosomal recessive condition (a prevalence of 2.6 cases/million) with childhood onset (AIRE gene) that, in addition to the classical triad (chronic candidiasis, adrenal insufficiency, and autoimmune hypoparathyroidism) may present with alopecia, vitiligo, and ATD [101]. AA, either patchy (most frequent), totalis, or universalis, is reported in APS-2, and APS-3, as well [102,103,104]. For instance, the rarest variant-AA universalis was recently identified as the onset of APS-3, before an ATD and type 1 diabetes mellitus diagnosis [103]. AA and vitiligo are regarded as “sister diseases” [104], one is due to an immune attack at the level of the hair follicle, the other, at the level of melanocytes. Also, psoriasis and lichen planus should be listed among dermatological illnesses with an immunologic component in other organs, including the thyroid [105,106]. A study of 158 patients with APS-1 showed a prevalence of 24% for alopecia, 17% for vitiligo, and 27.8% for ATD, respectively [107]. In addition to ATD, other AA comorbidities include asthma, atopic dermatitis, and allergic rhinitis. A recent study on 61,899 individuals with AA from 2012 to 2019 confirmed these associations. Of note, the prevalence of AA increased from 0.16% in 2012 to 0.27% in 2019, as an effect of modern society and autoimmunity issues [108].

### 4.4. Endocrine Perspective of Alopecia

Changing the perspective, a patient with alopecia may be first admitted to endocrinology, depending on the copresence of hormonal issues. Alopecia opens up several chapters. As mentioned, AA and ATD are reported together due to a common autoimmune background. Other thyroid conditions including abnormal hormones assays seem associated with TE, FFA, and LPP, as well as a few findings concerning thyroid cancer-associated AN with a low level of statistical evidence (Table 1) [13,16,17,18,19,21,25,29,30,33,34,39,40,41,42,43,44,45,46,47,48,49,50].

The assessments of thyroid hormones and antithyroid antibodies are mandatory in cases with particular aspects of AA (for instance, severe forms of AA, paediatric populations, patients with suspected or confirmed thyroid dysfunction, individuals with personal or familial medical history of autoimmune endocrine conditions like hypophysitis, chronic adrenal insufficiency, premature ovarian failure, type 1 diabetes mellitus, autoimmune hypoparathyroidism, polyglandular autoimmune syndromes, etc. or nonendocrine autoimmune comorbidities such as celiac disease, chronic autoimmune hepatitis, etc.), autoimmune TE, and LPP [109,110,111]. Hyperandrogenemia-related alopecia (associated or not with other clinical and biochemical elements of virilisation) is caused by androgen excess in females, of either an ovarian or adrenal cause like polycystic ovary syndrome (affecting 10% of women of reproductive age), ovarian tumours with virilisation potential, Cushing syndrome, congenital adrenal hyperplasia, etc. [112,113,114,115,116]. Anomalies of growth hormone signalling pathways, as seen in Laron syndrome, are associated with alopecia and also, rarely, hypopituitarism and hyperprolactinemia [84,117]. Among many other roles, vitamin D is a key player in trichology and skin metabolism through its local receptor; hypovitaminosis D might manifest with alopecia [118,119]. Loss of function in the vitamin D receptor (hereditary vitamin D-resistant type of rickets) associates hair loss with the paediatric onset and poor response to calcitriol treatment [120,121]. Some data showed a certain efficacy with regard to intralesional injections of vitamin D3 in AA [122]. When it comes to endocrine issues of a dermatologic approach concerning alopecia, especially AA, the topic and general administration of glucocorticoids is advised (selected cases) and traditional side effects of this medication should be taken into consideration [123,124].

Finally, an individual with alopecia may be admitted to a dermatologic and/or endocrine unit; and some aspects like personal medical history, family history in genetic conditions (like APS), and general phenotype (like myxoedema) are essential clues in order to decide the case management. Further studies are necessary to point out if a subgroup of patients with thyroid entities are prone to develop alopecia and, on the opposite, which is the protocol of thyroid panel assessment in subjects with alopecia without obvious signs of a thyroid disorder and, moreover, which is the extend of influencing alopecia amid hormonal dysfunction correction.

## 5. Conclusions

Overall, alopecia represents a very challenging condition for patients and physicians. A multidisciplinary team is required to improve the outcome and the quality of life. The common autoimmune background is suggestive of some types of alopecia and ATD. Yet, the underlying mechanisms are still a matter of debate. Non-AN types of alopecia (AA, FPHL, TE, FFA, or LPP) are studied in relationship with thyroid entities and some pointed out a higher prevalence than the general population, thus a state of awareness from a dual perspective, of trichology and endocrinology, is helpful. According to the data we have so far, a careful selection of patients with alopecia in order to address the thyroid evaluation depends on a multifactorial equation that includes dermatologic features (type of alopecia, other skin conditions with potential endocrine connections like vitiligo, etc.), the presence of hormonal anomalies, and of autoimmune background. Further, we need specific protocols of a dual perspective (dermatologic and hormonal) to help with the individual decisions of investigations and surveillance.

## Figures and Tables

**Table 1:** introduces the most clinically relevant study from a dermatologic perspective of alopecia and thyroid findings. The articles are displayed starting with 2013 (please see references [13,16,17,18,19,21,25,29,30,33,34,39,40,41,42,43,44,45,46,47,48,49,50]).

First Author (Reference)	Year of Publication	Type of Study	Studied Population	Type of Alopecia	Thyroid Involvement
Vincent [29]	2013	Prospective study	N = 176 patients with FPHL N1 = 1232 subjects (control group)	FPLH	Prevalence of thyroid dysfunction according to age groups: ≤20 y: 6.3% 21–40 y: 17.1% >40 y: 50%
Atanaskova Mesinkovska [45]	2014	Retrospective, case-control study	N = 166 patients with LPP N1 = 81 subjects (control group)	LPP	Thyroid involvement: 34% versus 11% (control) Hypothyroidism: 29% versus 9%
Famenini [25]	2015	Prospective study	N = 64 patients with FPHL (age: 20–88 y)	FPHL	Hypothyroidism: 31.25% No correlation between FPHL severity and thyroid disease
Miller [21]	2015	Retrospective study	N = 584 patients with AA N1= 172 subjects (control group)	AA	Thyroid involvement: 18.80% OR 2.84; 95%CI 1.55–5.18; *p* = 0.0004
Deo [34]	2016	Prospective study	N = 84 females with TE N1 = 135 females with non-TE alopecia (control group) (age: 15–60 y)	TE	Thyroid involvement: 17%, respective: hypothyroidism: 9.63% hyperthyroidism: 7.4%
Aldoori [43]	2016	Questionnaire-based study	N = 105 females with FFA (age: 42–79 y) N1 = 100 females (control group)	FFA	History of thyroid disease: 19% History of thyroid disease: 7%
Tolkachjov [44]	2017	Retrospective study	N = 7 males with FFA	FFA	None with thyroid involvement
Valesky [39]	2018	Meta-analysis	N = 932 patients with FFA (96.9% females)	FFA	Thyroid involvement: 31.4%
Brankov [48]	2018	Retrospective study	N = 334 patients with LLP N1 = 78 subjects with seborrheic dermatitis (control group)	LPP	Hashimoto’s thyroiditis: 6.3% versus 0%; *p* = 0.023 Hypothyroidism: 24.3% versus 12.8%; *p* = 0.028
Valesky [40]	2019	Retrospective, single-centre study	N = 12 females with FFA	FFA	Hypothyroidism: 58%
Lee [16]	2019	Meta-analysis	*n* = 87 studies	AA	ATD prevalence: 13.9% OR 1.66; 95% CI 0.82–3.38 Thyroid dysfunction: 12.5% OR 4.36; 95% CI 1.19–15.99 respective: subclinical hyperthyroidism: 5.7% OR 5.55; 95% CI 1.73–17.85 subclinical hypothyroidism: 10.4% OR 19.61; 95% CI 4.07–94.41
Kinoshita-Ise [17]	2019	Meta-analysis	N = 262,581 patients with AA N1 = 1,302,655 control subjects (AA free)	AA	Positive TPO-Ab: OR 3.58; 95% CI 1.96–6.53 Positive TG-Ab: OR 4.44; 95% CI 1.54–12.75 Risk of having both positive TPO-Ab and TG-Ab in the same subject: OR 2.32; 95% CI 1.08–4.98 Risk of having positive either of these antibodies in the same subject: OR 6.34; 95% CI 2.24–17.93 Positive TR-Ab: OR 60.90; 95% CI, 34.61–107.18
Paolino [13]	2019	Meta-analysis	N = 123 patients with AN	AN	Thyroid cancer: 7.3%
Kanti [41]	2019	Prospective study	N = 490 patients with FFA (95% females)	FFA	Thyroid involvement: 33.8% Females with thyroid involvement: 35% Males with thyroid involvement: 13%
Babahosseini [49]	2019	Retrospective study	N = 291 patients with LPP (age: 11–73 y)	LPP	Hypothyroidism: 10.58% (females), and 0.98% (males)
Xin [18]	2020	Meta-analysis	N = 2850 patients with AA N1 = 4667 subjects (control group)	AA	Thyroid disease: OR 3.66; 95% CI 2.90–4.61 Positive Tg-Ab: OR 3.83; 95% CI 1.92–7.63 Positive TPO-Ab: OR 4.07; 95% CI 2.66–6.22 Positive TM-Ab: OR 3.05; 95% CI 1.99–4.67 Hyperthyroidism: OR 1.43; 95% CI 0.36–5.57 Hypothyroidism: OR 4.07; 95% CI 1.95–8.49
Panchaprateep [42]	2020	Retroactive–prospective study	N = 51 females with FFA	FFA	Thyroid involvement: 15.7%
Larkin [46]	2020	Retrospective study	N = 232 females with LPP	LPP	Thyroid involvement: 30.1% Hypothyroidism: 23.3%
Manatis-Lornell [50]	2020	Retrospective study	N = 232 patients with LPP N1 = 192 subjects (control group)	LPP	Thyroid diseases (NOS): 28.4% versus 23.7% OR 1.28; 95% CI 0.83–1.98; *p* = 0.27 Thyroid nodules: 9.9% versus 11.3% OR 0.86; 95% CI 0.46–1.60; *p* = 0.63 Hypothyroidism: 20.7% versus 13.9% OR 1.61; 95% CI 0.96–2.70; *p* = 0.069 Hyperthyroidism: 1.7% versus 2.1% OR 0.83; 95% CI 0.21–3.38; *p* = 0.80 Goitre: 4.3% versus 6.7% OR 0.63; 95% CI 0.27–1.46; *p* = 0.28 Thyroiditis: 4.7% versus 4.1% OR 1.16; 95% CI 0.46–2.94; *p* = 0.76 Thyroid cancer: 1.3% versus 1.0% OR 1.26; 95% CI 0.21–7.60; *p* = 0.8
Dai [19]	2021	Retrospective study	N = 5929 patients with AA N1 = 59,290 subjects (control group)	AA	Increased risk of developing all thyroid diseases, including: toxic nodular goitre: aHR 10.17; 95% CI 5.32–19.44 nontoxic nodular goitre: aHR 5.23; 95% CI 3.76–7.28 thyrotoxicosis: aHR 7.96; 95% CI 6.01–10.54 Graves’s disease: aHR 8.36; 95% CI 5.66–12.35 thyroiditis: aHR 4.04; 95% CI 2.12–7.73 Hashimoto’s thyroiditis: aHR 4.35; 95% CI 1.88–10.04
Krysiak [30]	2021	Prospective study	N = 24 males (age: 18–40 y) with untreated autoimmune hypothyroidism and MPA N1= subjects (age: 18–40 y) with untreated autoimmune hypothyroidism without MPA (control group)	MPA	TPO-Ab [IU/mL; mean (SD)]: at the beginning of the study: 920 (301) versus 897 (315) (*p* = 0.0185) at the end of the study: 723 (256) versus 565 (227) (*p* = 0.0001) Tg-Ab [IU/mL; mean (SD)]: at the beginning of the study: 846 (358) versus 874 (242) (*p* = 0.0387) at the end of the study: 658 (243) versus 521 (219) (*p* < 0.0001) Free T4 [pmol/L; mean (SD)]: at the beginning of the study: 13.9 (2.5) versus 14.2 (2.3) (*p* = 0.0001) at the end of the study: 17.2 (2.9) versus 19.0 (3.0) (*p* < 0.0001) Free T3 [pmol/L; mean (SD)]: at the beginning of the study: 3.2 (0.6) versus 3.2 (0.5) (*p* = 0.0389) at the end of the study: 3.6 (0.7) versus 4.1 (0.8) (*p* < 0.0001)
Yorulmaz [33]	2021	Retrospective study	N = 3028 patients (age: 20–37 y) with TE	TE	Normal thyroid function: 94.3% Hypothyroidism: 3.9% Hyperthyroidism: 1.8%
Cantwell [47]	2021	Retrospective study	N = 16 males with LPP (age: 15.3–77.9 y)	LPP	Thyroid involvement: 15.8%

Abbreviations: AA = alopecia areata; AN = alopecia neoplastica; ATD = autoimmune thyroid disease; CI = confidence interval; FPHL = female pattern hair loss; FFA = frontal fibrosing alopecia; LLP = lichen planopilaris; HR = hazard ratio; IU = International units; MPA = male pattern alopecia; NOS = No other specified; OR = odds ratio; SD = standard deviation; TE = Telogen effluvium; TG-Ab = antityroglobulin antibodies; TM-Ab = anti-thrombomodulin antibodies; TPO-Ab = anti-thyroperoxidase antibodies; TR-Ab = TSH-receptor antibody; T3 = triiodothyronine; T4 = thyroxine; y = years; N = number of patients; N1 = control group; *n* = number of studies.

## Data Availability

Not applicable.

## Abbreviations

AA	alopecia areata
Ab	antibodies
AN	alopecia neoplastica
ATD	autoimmune thyroid disease
APS-1	autoimmune poly-endocrine syndrome type 1
CI	Confidence Interval
FFA	frontal fibrosing alopecia
FPHL	female pattern hair loss
HLA	Human Leukocyte Antigen
LPP	lichen planopilaris
IFN	interferon
IQR	interval inter-quartile
OR	Odds Ratio
NKG	Natural Killer Group
NOS	No other specified
MPA	male pattern alopecia
PPAR	peroxisome proliferator-activated receptor
SAVI	STING-associated vasculopathy with onset in infancy
TE	telogen effluvium
TH	thyroid hormones
TG-Ab	anti-thyroglobulin antibodies
TR-Ab	TSH-receptor antibody
TPO-Ab	antithyroperoxidase antibodies
TR	thyroid hormones receptor
T3	Triiodothyronine
T4	thyroxine
TSH	Thyroid Stimulating Hormone
